# Risk of Postoperative Infection in Total Knee Arthroplasty Patients with Preoperative Methicillin-Resistant *Staphylococcus aureus* (MRSA) Colonization

**DOI:** 10.3390/jcm14030765

**Published:** 2025-01-24

**Authors:** Aruni Areti, Terrul Ratcliff, Mehul M. Mittal, Dane K. Wukich, Antonia F. Chen, Senthil N. Sambandam

**Affiliations:** 1 Baylor College of Medicine, 1 Baylor Plz, Houston, TX 77030, USA; aruni.areti@bcm.edu; 2 UT Southwestern Medical Center, 5323 Harry Hines Blvd, Dallas, TX 75390, USA; terrul.ratcliff@utsouthwestern.edu (T.R.); dane.wukich@utsouthwestern.edu (D.K.W.); senthil.sambandam@utsouthwestern.edu (S.N.S.)

**Keywords:** methicillin-resistant *Staphylococcus aureus*, total knee arthroplasty, periprosthetic joint infection, surgical site infection, outcomes

## Abstract

**Background/Objectives:** While methicillin-resistant *Staphylococcus aureus* (MRSA) colonization is a known risk factor for surgical site infections, no definitive recommendations exist regarding preoperative *S. aureus* screening and decolonization protocols due to inconclusive evidence in Orthopedic Surgery. This study aimed to examine the correlation between preoperative MRSA colonization and postoperative infections in Total Knee Arthroplasty (TKA) patients. **Methods**: Data from 2005 to 2023 were collected from TriNetX, a global health research network, reviewing 237,360 unique patients. TKA patients were assigned under Current Procedural Terminology, while International Statistical Classification of Diseases Codes were used to identify preoperative comorbidities and postoperative complications. Demographic and analytical statistics were compared between MRSA-positive and control groups before and after propensity matching. **Results**: The MRSA-positive group had a significantly (*p* < 0.001) higher proportion of patients over 65 years (47.17% vs. 38.46%), obesity (41.76% vs. 34.67%), smoking disorders (33.36% vs. 19.73%), and diabetes (25.06% vs. 19.85%) compared to the control group. Postoperative complications were significantly (*p* < 0.001) more frequent in the MRSA-positive group, specifically periprosthetic joint infection (PJI) (4.11% vs. 0.79%, OR = 5.40), deep (0.12% vs. 0.01%, OR = 11.15) and superficial (0.37% vs. 0.09%, OR = 4.17) surgical site infections, and wound dehiscence (1.11% vs. 0.52%, OR = 2.13). The matched analysis confirmed significantly (*p* < 0.001) higher rates of PJI (4.39% vs. 1.18%, OR = 3.59). **Conclusions**: Our results illustrated preoperative colonization of MRSA as associated with an increased risk of wound-related complications. Surgeons and patients must consider preoperative MRSA colonization status when deciding if TKA is an optimal treatment option.

## 1. Introduction

Total knee arthroplasty (TKA) is among the most frequently performed orthopedic procedures, driven by an aging population and the increasing prevalence of osteoarthritis. It serves as the definitive treatment for severe knee joint degeneration, providing pain relief, restoring mobility, and enhancing quality of life [1]. The growing demand for TKA has also led to a projected 90% rise in revision TKA (rTKA) procedures by 2050, with estimates reaching 47,313 procedures [2]. A significant proportion of these revisions will be required due to periprosthetic joint infection (PJI), a serious complication diagnosed through a combination of clinical evaluation, positive cultures, sinus tract presence, and laboratory markers, including inflammatory indices per the Musculoskeletal Infection Society (MSIS) [3,4]. Such infections are often associated with substantial morbidity, mortality, and healthcare burden. PJI incidence ranges from 0.39% to 3.9%, with cumulative rates of 0.51% at 1 year, 1.12% at 5 years, and 1.65% at 15 years [5,6]. Notably, PJI is associated with significant mortality, with a 1-year mortality rate of approximately 4.33% and a fivefold increase in mortality at 1 year compared to aseptic revisions [7,8]. Given the increasing number of TKAs performed annually, optimizing infection prevention strategies is critical to improving patient outcomes.

*Staphylococcus aureus* (*S. aureus*) colonization poses a significant concern since *S. aureus* colonization has been linked to an increased risk of periprosthetic joint infection (PJI) or surgical site infection (SSI) in orthopedic surgery [9,10]. One-third of the population are potential carriers, and *S. aureus* is typically located in the anterior nares but can also be found on various skin surfaces as extranasal colonization [11]. One study found that methicillin-resistant *Staphylococcus aureus* (MRSA) was the organism present in 23% of primary total hip arthroplasty (THA) SSIs and 21% of revision THA SSIs [12]. Such infections can be challenging to treat and may result in unfavorable postoperative outcomes, such as increased mortality and longer hospital length of stay [12]. Moreover, the mortality risk linked to MRSA infection is twice as high as that of methicillin-sensitive *Staphylococcus aureus* (MSSA) infection, making MRSA especially concerning [13]. Understanding the severity of complications is essential for the risk-benefit analysis that patients and surgeons must consider when planning surgery. Such discussions can assist in patient optimization and targeted interventions that could reduce future postoperative complications [12].

At the 2018 International Consensus Meeting on Musculoskeletal Infection, no definitive recommendation was made regarding preoperative *S. aureus* screening and decolonization protocols due to inconclusive evidence [14] Notably, the absence of a clear statement on preoperative screening for periprosthetic joint infection (PJI) is not unique to the ICM criteria; similar omissions are present in other established guidelines, including those from the European Bone and Joint Infection Society (EBJIS) and the Musculoskeletal Infection Society (MSIS) [4,15]. Previous studies have shown that patients colonized with MRSA have a higher risk of developing invasive MRSA infections compared to those who are not colonized [16,17,18,19,20,21]. However, existing studies have yet to utilize a large population to assess the correlation between preoperative MRSA colonization and postoperative infection, especially among total knee arthroplasty (TKA) patients. This study evaluates the characteristics, demographics, and incidence of postoperative complications among primary TKA patients with preoperative MRSA colonization using a large, multi-institutional patient database (TriNetX) while applying propensity score matching for a more robust analysis. We hypothesize that MRSA-colonized primary TKA patients will have higher rates of postoperative complications compared to TKA patients without MRSA colonization.

## 2. Materials and Methods

### 2.1. Database Description

This retrospective cohort study utilized the TriNetX Research network (https://trinetx.com, Cambridge, MA, USA). The data used in this study were collected on 20 December 2023, from the TriNetX Research Network, which provided access to electronic medical records (diagnoses, procedures, medications, laboratory values, genomic information) from approximately 74 million patients from 82 U.S. healthcare organizations. TriNetX complies with the Health Insurance Portability and Accountability Act (HIPAA) and is ISO 27001:2013 certified, ensuring data protection and privacy [22,23].

### 2.2. Data Acquisition

This study was Institutional Review Board exempt as it involved a secondary analysis of de-identified data, with no human subject interaction or intervention, per Section 164.514(a) of the HIPAA Privacy Rule. MRSA screening practices in the United States involve a combination of active surveillance testing (AST), targeted screening, and decolonization protocols, particularly in acute-care hospitals and high-risk patient populations [24]. In this study, patients with Current Procedural Terminology (CPT) code 27447 (primary TKA) who had surgery between 2005 and 2023 were included. Those with diagnosis ICD-9 code (V02.54) and ICD-10 code (Z22.322) up to one year before TKA were classified as the preoperative MRSA colonization positive TKA group; others were the non-MRSA group.

Age, sex, obesity, diabetes, smoking, race, and ethnicity were analyzed in the demographics. Racial groups included White, Black or African American, Asian, American Indian or Alaska Native, Hispanic, and Unknown. Postoperative complications included blood loss anemia, acute renal failure (ARF), deep vein thrombosis (DVT), pulmonary embolism (PE), myocardial infarction (MI), pneumonia, cardiac arrest, and ventricular fibrillation (VF), and blood transfusion. Surgical complications included deep and superficial surgical site infections (SSI), wound dehiscence, prosthetic joint infection (PJI), dislocations, periprosthetic fractures, and mechanical complications. ICD-9 and ICD-10 codes listed in Appendix A were then used for the identification of preoperative comorbidities and postoperative complications.

### 2.3. Data Analysis

The date of TKA was designated as the index event from which the follow-up period began. Demographic information about patients was initially compiled using descriptive statistics. We carried out a matched and unmatched analysis. Using the preoperative variables, a 1:1 propensity matching algorithm was run. Propensity matching was conducted for age, sex, race, obesity, tobacco use, diabetes with complications, and diabetes without complications. *t*-tests were employed to examine continuous variables, while a chi-squared analysis was conducted to examine categorical variables. Fisher exact tests were applied when the incidence values were less than 5. For all tests, a *p*-value < 0.05 was regarded as statistically significant. A ratio of the incidence of medical and surgical complications in the MRSA colonized positive group to the incidence in the control group was reported as odds ratios, and their accompanying 95% confidence intervals (CIs).

### 2.4. Software Employed for Statistical Analysis and Data Visualization

Data compilation was performed using the TriNetx Live platform. Data management and statistical analysis were conducted using SPSS version 29 (IBM), while Microsoft Excel Version 16.93.1. was employed for additional statistical analysis and data visualization.

### 2.5. Data Integrity and Ethical Considerations

The TriNetx platform complies with HIPAA and GDPR regulations, ensuring data security and patient privacy by using only de-identified, aggregate data. This study was Institutional Review Board exempt as it involved a secondary analysis of de-identified data, with no human subject interaction or intervention, per Section 164.514(a) of the HIPAA Privacy Rule.

## 3. Results

### 3.1. Demographics

A total of 237,360 patients who had undergone primary TKA were identified using the TriNetX database during the study period. Among this cohort, 1628 patients (0.7%) tested positive for MRSA, serving as the study group. The remaining 235,732 patients were identified as the control group. The demographic data for these groups are included in Table 1.

The MRSA-positive group had a significantly higher proportion of patients >65 years (47.17%) compared to the control group (38.46%) (*p* < 0.001). The proportion of women was similar (*p* = 0.782) between the MRSA-positive (60.59%) and control groups (60.91%).

The MRSA-positive group was significantly more likely to be obese (41.76%) compared to the control group (34.67%) (*p* < 0.001). Smoking disorders were more common in the MRSA-positive group (33.36%) compared to the control group (19.73%) (*p* < 0.001). The MRSA-positive group had a significantly higher rate of diabetes without complications (25.06%) than patients in the control group (19.85%) (*p* < 0.001).

The MRSA-positive group had a larger proportion of individuals identified as Other Race (2.58% vs. 2.43%), American Indians or Alaska Natives (0.31% vs. 0.27%), White (76.64% vs. 74.84%), and Hispanic or Latino individuals (10.63% vs. 10.46%). The control group had a higher proportion of Asians (2.07% vs. 0.86%) and Black or African Americans (9.74% vs. 8.78%). All differences were statistically significant (*p* = 0.029).

### 3.2. Unmatched Analysis 30-Day Post-Operative Outcomes

The unmatched analysis comparing postoperative complications between TKA MRSA carrier patients and the control group is summarized in Table 2. Deep SSI was significantly higher (OR = 11.15 [95% CI: 2.64–47.02], *p* < 0.001) in the MRSA-positive group (0.12%) compared to the control group (0.01%). Superficial SSI was also more frequent (OR = 4.17 [95% CI: 1.85–9.40], *p* < 0.001) among MRSA-positive patients (0.37%) versus the control group (0.09%). Wound dehiscence occurred more frequently (OR = 2.13 [95% CI: 1.34–3.41], *p* < 0.001) in the MRSA-positive group (1.11%) versus the control group (0.52%), and PJI rates were higher (OR = 5.40 [95% CI: 4.21–6.93], *p* < 0.001) in MRSA-positive TKA patients (4.11%) versus control patients (0.78%).

Other significant differences included acute renal failure, which occurred in 3.38% of the MRSA-positive patients compared to 1.95% in control patients (OR = 1.75 [95% CI: 1.34–2.29], *p* < 0.001). Blood loss anemia was also higher (OR = 1.93 [95% CI: 1.66–2.25], *p* < 0.001) in MRSA-positive patients (11.86%) versus control patients (6.46%). Pneumonia was significantly more common (OR = 2.31 [95% CI: 1.53–3.50], *p* < 0.001) in the MRSA-positive group (1.41%) compared to the control group (0.61%). Myocardial infarction was also more prevalent (OR = 2.36 [95% CI: 1.17–4.75], *p* = 0.013) in the MRSA-positive group (0.49%) versus the control group (0.21%). Pulmonary embolism was significantly more frequent (OR = 1.55 [95% CI: 1.00–2.38], *p* = 0.047) in the MRSA-positive group (1.29%) compared to the control group (0.83%). Finally, stroke was significantly more frequent (OR = 2.10 [95% CI: 1.69–2.60], *p* < 0.001) in the MRSA-positive group (5.47%) compared to the control group (2.67%).

### 3.3. Matched Analysis 30-Day Post-Operative Outcomes

The matched analysis of postoperative complications between MRSA-positive patients and the control group is summarized in Table 3. While deep SSI was more frequent (OR = 1.88 [95% CI: 0.17–20.69], *p* = 0.60) in the MRSA-positive group (0.12%) compared to the control group (0.07%), and superficial SSI was greater (OR = 1.41 [95% CI: 0.39–4.99], *p* = 0.60) in MRSA-positive patients (0.37%) compared to control patients (0.26%), these differences were not statistically significant.

On the other hand, PJI (MRSA 4.12% vs. control 1.18%, OR = 3.59 [95% CI: 2.12–6.07], *p* < 0.001), stroke (MRSA 5.47% vs. control 2.89%, OR = 1.95 [95% CI: 1.35–2.81], *p* < 0.001), and blood loss anemia (MRSA 11.86% vs. control 6.89%, OR = 1.82 [95% CI: 1.41–2.33], *p* < 0.001) were significantly higher in the MRSA-positive group compared to the control group. Wound dehiscence in the MRSA-positive group was higher (OR = 1.88 [95% CI: 0.84–4.21], *p* = 0.12) at 1.11% versus the control group (0.59%), though this difference was not statistically significant.

ARF (MRSA 3.38% vs. control 1.84%, OR = 1.87 [95% CI: 1.18–2.96], *p* = 0.007) and pulmonary embolism (MRSA 1.29% vs. control 0.59%, OR = 2.20 [95% CI: 1.01–4.82], *p* = 0.043) were both significantly higher in MRSA-positive TKA patients versus control patients. Myocardial infarction was also significantly more prevalent (OR = 7.53 [95% CI: 0.94–60.24], *p* = 0.025) in the MRSA-positive group (0.49%) compared to the control group (0.07%).

## 4. Discussion

*S. aureus* is the most commonly identified organism in orthopedic SSI infections and is responsible for approximately 63% of SSI after TKA [11]. Generally, it has been established that MRSA/MSSA colonization is associated with an increased risk of subsequent SSI, but evidence on preoperative *S. aureus* screening and decolonization remains inconclusive [13,14,20].

Our study categorized SSI into four major wound-related complications: deep SSI, superficial SSI, wound dehiscence, and PJI. In the matched analysis, PJI remained significantly higher in the MRSA-positive group, while wound dehiscence and deep and superficial SSI were not statistically significant. Both analyses demonstrated significantly higher rates of blood loss anemia, PJI, stroke, and pneumonia in the MRSA-positive group. In the unmatched analysis, all four wound-related complications—deep SSI, superficial SSI, wound dehiscence, and PJI—were significantly associated with MRSA positivity. These findings support our initial hypothesis that MRSA-colonized primary TKA patients would have higher rates of postoperative complications compared to non-colonized patients.

MRSA colonization and its association with postoperative complications is not unique to TKA. Nguyen et al. found patients with prior MRSA colonization or infection had 9–10 times greater odds of MRSA SSI [25]. Kalra et al. and Sing et al. both determined that MRSA-positive patients had increased odds of developing SSI postoperatively [17,19]. We demonstrated that MRSA colonization before TKA is associated with an increased risk of deep and superficial SSI in the unmatched analysis, but this difference was not significant in the matched analysis. Differences in the unmatched analysis may be due to confounding factors controlled for in the matched analysis. It is well recognized in adult reconstructive surgery that obesity, active tobacco use, and diabetes are associated with increased risks of SSI; therefore, controlling for these variables is a potential explanation for differences in findings based on the matched analysis [26]. The differences observed between the unmatched and matched analyses highlight the potential for residual confounding in unmatched comparisons, which may overestimate associations due to underlying patient factors that are not controlled for. Despite controlling for obesity, active tobacco use, and diabetes, PJI was significantly associated with our MRSA-positive group.

Therefore, surgeons must consider patients’ preoperative MRSA colonization status when counseling them on the risks of TKA, especially in the context of common comorbidities that may exacerbate the risk of SSI and PJI. We observed a statistically significant higher percentage of patients in the MRSA-positive group who were obese (BMI > 30), smokers, and diabetic compared to the control group. Previous research shows that obese orthopedic surgery patients have double the risk of developing SSI, and obese TKA patients are 6.7 times more likely to experience PJI [12,27]. Patients with diabetes experience higher antimicrobial resistance and co-infection rates and are more susceptible to MRSA infection [28]. Smoking increases mucus production, impairs epithelial elastic properties, and decreases IgA production. These changes facilitate bacterial colonization in the nasopharynx, ultimately leading to smokers having higher rates of MRSA colonization than nonsmokers [29].

Currently, the average age of patients undergoing TKA is between 65 and 67 years [30]. Our data demonstrated a significantly higher prevalence of patients over 65 years in the MRSA-positive group. This is relevant since such an age group is more susceptible to risk factors such as obesity and diabetes and has longer hospital lengths of stay (LOS), an independent risk factor for PJI [12,31,32]. While older adults have an inherently higher MRSA risk due to immune senescence and healthcare exposure, diabetes further exacerbates this risk through impaired immune function, increased antimicrobial resistance, and poor wound healing. They are also known to experience overall increased postoperative complications, admission to the intensive care unit (ICU), and discharge to a skilled care facility [30]. Elderly patients’ increased risk of MRSA colonization is likely multifactorial, related to their age-related weakened immune system, chronic illnesses, potential close living quarters (if living in a nursing home), and recent visits to other healthcare facilities [33].

Xiang et al. found that patients over 60 years old who underwent surgery faced an independent risk factor for postoperative pneumonia [34]. This is consistent with our findings, as both our matched and unmatched analyses identified pneumonia as a significant complication in the MRSA-positive group. MRSA is well-established as the leading cause of hospital-acquired and ventilator-associated pneumonia, which is also known as postoperative pneumonia [35]. In the context of surgical trauma, the breakdown of the body’s skin and tissues compromises the first line of immune defense, providing opportunities for bacterial infections such as pneumonia [34]. Although MRSA colonization may be asymptomatic, it can lead to infections if the balance between host defense and bacterial virulence shifts in favor of the bacteria due to surgical trauma [36].

Such infections predispose patients to an inflammatory response in the body, which may result in anemia or chronic inflammation. The risk of anemia may be exacerbated by MRSA utilizing the host’s iron to survive and grow, potentially putting patients at risk for iron deficiency [37]. Gram-positive bacteria, such as *S. aureus*, also produce hemolysins and have membrane receptors that scavenge and bind myoglobin, hemoglobin, and hemoglobin-haptoglobin complexes [38]. Our results support this, revealing significant associations between MRSA-positive status and blood loss anemia in unmatched and matched analyses.

Our study found a significant relationship between stroke and the MRSA-positive cohort. This may be due to MRSA leading to *Staphylococcus aureus* bacteremia (SAB), which increases stroke risk for up to 180 days [39]. *S. aureus* causes 30% of infectious endocarditis cases, which involves inflammation of the endocardium and valves [40]. This often results in ischemic strokes, the most common neurological complication, with stroke risk beginning four months before and up to five months after diagnosis [40,41]. The mechanism by which infection causes stroke remains inconclusive. A theory is that stimulating the inflammatory response leads to alterations in platelet aggregation and vasodilation dysfunction [42].

Historically, vancomycin has been the primary treatment of choice for drug-resistant Gram-positive infections, such as MRSA [43]. However, Buis et al. found vancomycin therapy to be independently associated with ARF in SAB patients, as noted in other studies [44,45]. Patients in our MRSA-positive group had a higher risk of developing ARF. In some cases, combining aminoglycosides with vancomycin is used as an alternative, but it increases the risk of nephrotoxicity and ototoxicity. This is important, particularly in hospitals like the one described by Kalra et al., which consider decolonization or preoperative vancomycin for MRSA-positive patients undergoing high-risk surgeries like TKA [19]. We could not clarify specific antibiotic treatments or decolonization protocols due to the nature of our database results; however, these forms of treatment or decolonization protocols could explain why our study illustrated a significantly increased risk of ARF in MRSA-positive patients. Regional differences in MRSA screening vary by geography, socioeconomic status, and healthcare facility resources. A study on racial disparities found that socioeconomic factors largely explained the higher MRSA incidence among Black individuals compared to White individuals, with lower SES as a key mediator [46]. Hospital complexity and location also impact screening—urban hospitals typically have comprehensive MRSA programs, while rural hospitals, with fewer resources, screen less frequently [47]. An Ohio nursing home study found MRSA prevalence was highest in urban sites (36.5%) compared to suburban (16.9%) and rural (0%) sites [48].

The clinical utility and cost-effectiveness of universal MRSA screening for all TKA patients remain debated. Suratwala et al. found that universal pre-admission MRSA screening in total joint arthroplasty patients did not significantly reduce infection rates and increased costs, raising concerns about the value of routine testing [49]. However, in high-risk settings, particularly urban hospitals with higher MRSA prevalence, universal screening may be more justified. Hubben et al. demonstrated that in such environments, universal screening was more cost-effective due to the higher risk of MRSA transmission [50]. A balanced approach could involve risk-stratified screening, where patients with established risk factors such as those highlighted in our study undergo testing and decolonization, while universal screening is reserved for high-MRSA burden institutions.

### Limitations

A limitation of our retrospective study was the inability to determine the proportion of patients screened for MRSA. Unlike standardized national guidelines in some countries, MRSA screening in the U.S. is often institution-specific. Individual healthcare institutions determine their own protocols based on risk stratification models. Some hospitals implement universal screening for surgical patients, while others screen only high-risk individuals (e.g., patients with a history of MRSA colonization, recent hospitalizations, dialysis patients, or those with prolonged healthcare exposure) [24,51]. This study could not account for specific screening protocols across the 82 included healthcare organizations, as TriNetX does not provide data on institutional MRSA screening policies. This variability may have influenced our findings, as the control group consisted of patients without a documented MRSA-positive screening, but some may not have been screened at all.

We also do not know if MRSA-positive patients were successfully decolonized, which could have affected our results and analysis. The effectiveness and approach of decolonization on postoperative outcomes, particularly in orthopedic patients, remain uncertain. Ammerlaan et al. suggested mupirocin, whereas Karla et al. approached decolonization with preoperative vancomycin [10,37]. Limited research has gone on to compare either approach’s effectiveness. This makes it challenging to recommend clear instructions for screening and decolonization.

There are inherent limitations in a retrospective study utilizing TriNetX. Using data only from participating HCOs excludes data from follow-up treatment at non-network HCOs or loss to follow-up. All participating HCOs are from within the United States, which cannot be generalized globally. However, other international studies have demonstrated an increased risk of SSI in MRSA-colonized patients [13,52]. Electronic health record systems have limited standardization and large degrees of variability across users and organizations. For example, inaccuracies in ICD coding may stem from clerical errors and billing or coding staff omitting comorbidity codes due to perceived irrelevance. Overall, ICD codes cannot capture all patient details, limiting their ability to fully represent a population’s characteristics [53]. For example, infection classification in this study was based on ICD coding rather than standardized clinical criteria, which may not fully capture diagnostic nuances. While coding differentiates SSI and PJI, it does not account for variations in institutional diagnostic practices, which could lead to misclassification. In addition, given the retrospective design, our findings establish associations rather than causality, and further prospective studies are needed to confirm these relationships.

Lastly, our study did not include American Society of Anesthesiologists (ASA) scores or other validated preoperative risk stratification tools, which could have provided further insight into the overall health status of MRSA-positive patients. While propensity matching adjusted for key comorbidities such as obesity, smoking, and diabetes, unmeasured confounders such as frailty, functional status, or perioperative hemodynamic factors may have contributed to observed associations between MRSA colonization and complications like myocardial infarction and pulmonary embolism.

## 5. Conclusions

Our results demonstrated that preoperative MRSA colonization in primary TKA patients was associated with an increased risk of subsequent wound-related complications, including deep SSI and superficial SSI in the unmatched analysis and PJI and wound dehiscence in both the unmatched and matched analyses. Among our demographic and admission characteristics, four major risk factors—age > 65 years, diabetes, obesity, and smoker status—were significantly correlated with the MRSA-positive group. This study demonstrates that preoperative MRSA colonization status may help surgeons identify patients at higher risk for wound complications, soft tissue infection, and PJI after TKA.

## Figures and Tables

**Table 1:** Patient Demographic and Admission Characteristics of methicillin-resistant *Staphylococcus aureus* (MRSA) positive (+) patients and Control patients.

Variables		MSRA (+) (*n* = 1628)	Control Group (*n* = 235,732)	*p*-Value
Age > 65		768 (47.17%)	90,651 (38.46%)	<0.001 *
Sex (proportion of women)		924 (60.59%)	138,082 (60.91%)	0.782
Obesity		680 (41.76%)	81,733 (34.67%)	<0.001 *
Smoking disorder		543 (33.36%)	46,528 (19.73%)	<0.001 *
Diabetes without complications		408 (25.06%)	46,780 (19.85%)	<0.001 *
Race				
	American Indian or Alaska Native	5 (0.31%)	639 (0.27%)	0.029 *
	Asian	14 (0.86%)	4887 (2.07%)	0.029 *
	Black or African American	143 (8.78%)	22,958 (9.74%)	0.029 *
	Pacific Islander	3 (0.18%)	476 (0.20%)	0.029 *
	Other Race	42 (2.58%)	5729 (2.43%)	0.029 *
	Hispanic	173 (10.63%)	24,654 (10.46%)	0.029 *
	White	1248 (76.64%)	176,389 (74.84%)	0.029 *

* *p* < 0.05. MRSA (+) = Postive preoperative colonization or infection with MRSA.

**Table 2:** Unmatched Analysis: Postoperative complications differences between Characteristics of methicillin-resistant *Staphylococcus aureus* (MRSA) positive (+) patients and Control patients.

Postoperative Variables	MSRA (+) (*n* = 1628)	Control Group (*n* = 237,360)	Odds Ratio (CU/NCU Group)	Odds Ratio 95% Confidence Interval	*p*-Value
Deep SSI	2 (0.12%)	26 (0.01%)	11.15	(2.64, 47.02)	<0.001 *
Wound Dehiscence	18 (1.11%)	1228 (0.52%)	2.13	(1.34, 3.41)	<0.001 *
Periprosthetic Infection	67 (4.11%)	1859 (0.78%)	5.4	(4.21, 6.93)	<0.001 *
Superficial SSI	6 (0.37%)	209 (0.09%)	4.17	(1.85, 9.40)	<0.001 *
Acute Renal Failure	55 (3.38%)	4619 (1.95%)	1.75	(1.34, 2.29)	<0.001 *
Blood Loss Anemia	193 (11.86%)	15,343 (6.46%)	1.93	(1.66, 2.25)	<0.001 *
Cardiac Arrest and VF	1 (0.06%)	106 (0.04%)	1.37	(0.19, 9.80)	0.755
DVT	27 (1.66%)	2986 (1.26%)	1.32	(0.90, 1.93)	0.159
Periprosthetic Mechanical Complication	10 (0.61%)	829 (0.35%)	1.75	(0.94, 3.27)	0.075
Myocardial Infarction	8 (0.49%)	493 (0.21%)	2.36	(1.17, 4.75)	0.013 *
Pulmonary Embolism	21 (1.29%)	1977 (0.83%)	1.55	(1.00, 2.38)	0.047 *
Periprosthetic Fracture	7 (0.43%)	551 (0.23%)	1.84	(0.87, 3.89)	0.103
Pneumonia	23 (1.41%)	1452 (0.61%)	2.31	(1.53, 3.50)	<0.001 *
Blood Transfusion	1 (0.06%)	104 (0.04%)	1.39	(0.19, 9.99)	0.741
Stroke	89 (5.47%)	6325 (2.67%)	2.1	(1.69, 2.60)	<0.001 *

* *p* < 0.05. SSI = Surgical Site Infection; VF = Ventricular Fibrillation; DVT = Deep Vein Thrombosis. MRSA (+) = Postive preoperative colonization or infection with MRSA.

**Table 3:** Matched Analysis: Postoperative complications differences between Characteristics of methicillin-resistant *Staphylococcus aureus* (MRSA) positive (+) patients and Control patients.

Postoperative Variables	MRSA (+) (*n* = 1628)	Control Group (*n* = 1525)	Odds Ratio (CU/NCU Group)	Odds Ratio 95% Confidence Interval	*p*-Value
Deep SSI	2 (0.12%)	1 (0.07%)	1.88	(0.17, 20.69)	0.602
Wound Dehiscence	18 (1.11%)	9 (0.59%)	1.88	(0.84, 4.21)	0.116
Periprosthetic Infection	67 (4.12%)	18 (1.18%)	3.59	(2.12, 6.07)	<0.001 *
Superficial SSI	6 (0.37%)	4 (0.26%)	1.41	(0.39, 4.99)	0.596
Acute Renal Failure	55 (3.38%)	28 (1.84%)	1.87	(1.18, 2.96)	0.007 *
Blood Loss Anemia	193 (11.86%)	105 (6.89%)	1.82	(1.41, 2.33)	<0.001 *
Cardiac Arrest and VF	1 (0.06%)	0 (0.0%)	0.52	(0.05, 14.99)	0.333
DVT	27 (1.66%)	15 (0.98%)	1.7	(0.90, 3.20)	0.099
Periprosthetic Mechanical Complication	10 (0.61%)	3 (0.2%)	3.14	(0.86, 11.41)	0.067
Myocardial Infarction	8 (0.49%)	1 (0.07%)	7.53	(0.94, 60.24)	0.025
Pulmonary Embolism	21 (1.29%)	9 (0.59%)	2.2	(1.01, 4.82)	0.043
Periprosthetic Fracture	7 (0.43%)	3 (0.2%)	2.19	(0.57, 8.49)	0.244
Pneumonia	23 (1.41%)	13 (0.85%)	1.67	(0.84, 3.30)	0.139
Blood Transfusion	1 (0.06%)	1 (0.07%)	0.94	(0.06, 14.99)	0.963
Stroke	89 (5.47%)	44 (2.89%)	1.95	(1.35, 2.81)	<0.001 *

* *p* < 0.05. SSI = Surgical Site Infection; VF = Ventricular Fibrillation; DVT = Deep Vein Thrombosis. MRSA (+) = Postive preoperative colonization or infection with MRSA.

## Data Availability

The data that support the findings of this study are available from the corresponding author, A.A., upon reasonable request.

## Appendix A

### Appendix A.1. CPT Codes for Procedures

TKA: 27447

### Appendix A.2. Complications and Corresponding ICD 9 and 10 Codes

Stroke: 436, 4370, 4371, 4380, 43810, 43811, 43812, 43813, 43814, 43819, 43820, 43821, 43822, 43830, 43831, 43832, 43840, 43841, 43842, 43850, 43851, 43852, 43853, 4386, 4387, 43881, 43882, 43883, 43884, 43885, 43889, 4389, V1254, Z8673, G450, G451, G452, G453, G454, G458, G459, G460, G462, G463, G464, G465, G466, G467, G468, I6300, I63011, I63012, I63013, I63019, I6302, I63031, I63032, I63033, I63039, I6309, I6310, I63111, I63112, I63113, I63119, I6312, I63131, I63132, I63133, I63139, I6319, I6320, I63211, I63212, I63213, I63219, I6322, I63231, I63232, I63233, I63239, I6329, I6330, I63311, I63312, I63313, I63319, I63321, I63322, I63323, I63329, I63331, I63332, I63333, I63339, I63341, I63342, I63343, I63349, I6339, I6340, I63411, I63412, I63413, I63419, I63421, I63422, I63423, I63429, I63431, I63432, I63433, I63439, I63441, I63442, I63443, I63449, I6349, I6350, I63511, I63512, I63513, I63519, I63521, I63522, I63523, I63529, I63531, I63532, I63533, I63539, I63541, I63542, I63543, I63549, I6359, I636, I638, I6381, I6389, I639, I6930, I6931, I69310, I69311, I69312, I69313, I69314, I69315, I69318, I69319, I69320, I69321, I69322, I69323, I69328, I69331, I69332, I69333, I69334, I69339, I69341, I69342, I69343, I69344, I69349, I69351, I69352, I69353, I69354, I69359, I69361, I69362, I69363, I69364, I69365, I69369, I69390, I69391, I69392, I69393, I69398, I6980, I6981, I69810, I69811, I69812, I69813, I69814, I69815, I69818, I69819, I69820, I69821, I69822, I69823, I69828, I69831, I69832, I69833, I69834, I69839, I69841, I69842, I69843, I69844, I69849, I69851, I69852, I69853, I69854, I69859, I69861, I69862, I69863, I69864, I69865, I69869, I69890, I69891, I69892, I69893, I69898, I6990, I6991, I69910, I69911, I69912, I69913, I69914, I69915, I69918, I69919, I69920, I69921, I69922, I69923, I69928, I69931, I69932, I69933, I69934, I69939, I69941, I69942, I69943, I69944, I69949, I69951, I69952, I69953, I69954, I69959, I69961, I69962, I69963, I69964, I69965, I69969, I69990, I69991, I69992, I69993, I69998
Cardiac Arrest and VF: I4901, 4275, 42741
Heart Failure: I509, 428, 4281, 4282, 42821, 42822, 42823, 4283, 42831, 42832, 42833, 4284, 42841, 42842, 42843, 4289, 40201, 40211, 40291, 40401, 40403, 40411, 40413, 40491, 40493
Cardiac Arrhythmia: I49, 4279, 42789, 4269, 42760, 9971, 3062, 7802
Acute Renal Failure: N170, N171, N172, N178, N179, 584, 5945, 5946, 5847, 5848, 5849
Myocardial Infarction: I2101, I2102, I2111, I2113, I2114, I2119, I2121, I2129, I21A1, 410, 4100, 41000, 41001, 41002, 4101, 41010, 41011, 41012, 4102, 41020, 41021, 41022, 4103, 41030, 41031, 41032, 4104, 41040, 41041, 41042, 4105, 41050, 41051, 41052, 4106, 41060, 41061, 41062, 4107, 41070, 41071, 41072, 4108, 41080, 41081, 41082, 4109, 41090, 41091, 41092
Blood Loss Anemia: D62, 2800, 2878, 2879
Pneumonia: J189, J159, J22, 486, 481, 485, 4870, 4871, 4878, 480, 4800, 4801, 4802, 4803, 4808, 4809, 482, 4820, 4821, 4822, 4823, 48230, 48231, 48232, 48239, 4824, 48240, 48241, 48242, 48249, 4828, 48281, 48282, 48283, 48284, 48289, 4829, 483, 4830, 4831, 4838, 484, 4841, 4843, 4845, 4846, 4847, 4848, 488, 4880, 48801, 48802, 48809, 4881, 48811, 48812, 48819, 4888, 48881, 48882, 48889
Blood Transfusion: 30233N1, 990, 9900, 9901, 9902, 9903, 9904, 9905, 9906, 9907, 9908, 9909
Pulmonary Embolism: I2602, I2609, I2692, I2699, 4151, 41511, 41512, 41513, 41519
Deep Vein Thrombosis (DVT): I82401, I82402, I82403, I82409, I82411, I82412, I82413, I82419, I82421, I82422, I82423, I82429, I82431, I82432, I82433, I82439, I82441, I82442, I82443, I82491, I82492, I82493, I82499, I824Y1, I824Y2, I824Y3, I824Y9, I824Z1, I824Z2, I824Z3, I824Z4, 451, 4510, 4511, 45111, 45119, 4512, 4518, 45181, 45182, 45183, 45184, 45189, 4519
Periprosthetic Fracture: T84010A, T84011A, T84012A, T84013A, T84018A, T84019A, M9665, M96661, M96662, M96669, M96671, M96672, M96679, M9669, M9701XA, M9702XA, M9711XA, M9712XA, 99644
Periprosthetic Dislocation: T84020A, T84021A, T84022A, T84023A, T84028A, T84029A, 99642
Periprosthetic Mechanical Complication: T84090A, T84091A, T84092A, T84093A, T84098A, T84099A, 99639, 9964, 99640, 99641, 99643, 99645, 99646, 99647, 99649
Periprosthetic Infection: T8450XA, T8451XA, T8452XA, T8453XA, T8459XA, T8454XA, 99666, 99667, 99669
Superficial SSI: T8141XA
Deep SSI: T8142XA
Wound Dehiscence: T8130XA, T8131XA, T8132XA, 99831, 99832

### Appendix A.3. Comorbidities and Corresponding ICD 9 and 10 Codes

Pre-operative MRSA colonization: V02.54 & Z22.322
Stroke: 430, 431, 4320, 4321, 4329, 43301, 43311, 43321, 43331, 43381, 43391, 43401, 43411, 43491, 4350, 4351, 4352, 4353, 4358, 4359, 436, 4370, 4371, 4372, 4373, 4374, 4375, 4376, 4377, 4378, 4379, I60, I600, I6000, I6001, I6002, I6009, I601, I6010, I6011, I6012, I6019, I602, I6020, I6021, I6022, I6029, I603, I6030, I6031, I6032, I6039, I604, I6040, I6041, I6042, I6049, I605, I6050, I6051, I6052, I6059, I606, I6060, I6061, I6062, I6069, I607, I6070, I6071, I6072, I6079, I608, I6080, I6081, I6082, I6089, I609, I6090, I6091, I6092, I6099, I61, I610, I611, I612, I613, I614, I615, I616, I618, I619, I620, I6200, I6201, I6202, I6203, I6209, I621, I6210, I6211, I6212, I6213, I6219, I629, I63, I630, I6300, I6301, I6302, I6303, I6309, I631, I6310, I6311, I6312, I6313, I6319, I632, I6320, I6321, I6322, I6323, I6329, I633, I6330, I6331, I6332, I6333, I6339, I634, I6340, I6341, I6342, I6343, I6349, I635, I6350, I6351, I6352, I6353, I6359, I636, I638, I6381, I6382, I6389, I639, I64, I650, I651, I652, I653, I658, I659, I66, I660, I661, I662, I663, I668, I669, I67, I670, I671, I672, I673, I674, I675, I676, I677, I678, I679, I688, I698, I699, I700, I701, I702, I703, I708, I709, I72, I720, I721, I722, I728, I729, I73, I730, I731, I732, I733, I738, I739, I74, I740, I741, I742, I743, I744, I745, I748, I749, I75, I750, I751, I752, I753, I758, I759, I76, I77, I770, I771, I772, I773, I774, I775, I776, I777, I778, I779, I78, I780, I781, I782, I783, I784, I785, I786, I787, I788, I789, I79, I790, I791, I792, I793, I794, I795, I796, I797, I798, I799
Smoking: 3051, 30510, 30511, 30512, 30513, 30514, 30515, 30516, 30517, 30518, 30519, 64900, 64901, 64902, 64903, 64904, 98984, F17200, F17201, F17203, F17208, F17209, F17210, F17211, F17213, F17218, F17219, F17220, F17221, F17223, F17228, F17229, F17290, F17291, F17293, F17298, F17299, O99330, O99331, O99332, O99333, O99334, O99335, O99336, Z720, Z87791
Diabetes: 25000, 25001, 25002, 25003, 25010, 25011, 25012, 25013, 25020, 25021, 25022, 25023, 25030, 25031, 25032, 25033, 25040, 25041, 25042, 25043, 25050, 25051, 25052, 25053, 25060, 25061, 25062, 25063, 25070, 25071, 25072, 25073, 25080, 25081, 25082, 25083, 25090, 25091, 25092, 25093, E0800, E0801, E0802, E0803, E0804, E0805, E0806, E0807, E0808, E0809, E0810, E0811, E0812, E0813, E0814, E0815, E0816, E0817, E0818, E0819, E0820, E0821, E0822, E0823, E0824, E0825, E0826, E0827, E0828, E0829, E0830, E0831, E0832, E0833, E0834, E0835, E0836, E0837, E0838, E0839, E0840, E0841, E0842, E0843, E0844, E0845, E0846, E0847, E0848, E0849, E0850, E0851, E0852, E0853, E0854, E0855, E0856, E0857, E0858, E0859, E0860, E0861, E0862, E0863, E0864, E0865, E0866, E0867, E0868, E0869, E0870, E0871, E0872, E0873, E0874, E0875, E0876, E0877, E0878, E0879, E0880, E0881, E0882, E0883, E0884, E0885, E0886, E0887, E0888, E0889, E089, E0900, E0901, E0902, E0903, E0904, E0905, E0906, E0907, E0908, E0909, E0910, E0911, E0912, E0913, E0914, E0915, E0916, E0917, E0918, E0919, E0920, E0921, E0922, E0923, E0924, E0925, E0926, E0927, E0928, E0929, E0930, E0931, E0932, E0933, E0934, E0935, E0936, E0937, E0938, E0939, E0940, E0941, E0942, E0943, E0944, E0945, E0946, E0947, E0948, E0949, E0950, E0951, E0952, E0953, E0954, E0955, E0956, E0957, E0958, E0959, E0960, E0961, E0962, E0963, E0964, E0965, E0966, E0967, E0968, E0969, E0970, E0971, E0972, E0973, E0974, E0975, E0976, E0977, E0978, E0979, E0980, E0981, E0982, E0983, E0984, E0985, E0986, E0987, E0988, E0989, E0990, E0991, E0992, E0993, E0994, E0995, E0996, E0997, E0998, E0999, E1000, E1001, E1002, E1003, E1004, E1005, E1006, E1007, E1008, E1009, E1010, E1011, E1012, E1013, E1014, E1015, E1016, E1017, E1018, E1019, E1020, E1021, E1022, E1023, E1024, E1025, E1026, E1027, E1028, E1029, E1030, E1031, E1032, E1033, E1034, E1035, E1036, E1037, E1038, E1039, E1040, E1041, E1042, E1043, E1044, E1045, E1046, E1047, E1048, E1049, E1050, E1051, E1052, E1053, E1054, E1055, E1056, E1057, E1058, E1059, E1060, E1061, E1062, E1063, E1064, E1065, E1066, E1067, E1068, E1069, E1070, E1071, E1072, E1073, E1074, E1075, E1076, E1077, E1078, E1079, E1080, E1081, E1082, E1083, E1084, E1085, E1086, E1087, E1088, E1089, E1090, E1091, E1092, E1093, E1094, E1095, E1096, E1097, E1098, E1099, E1100, E1101, E1102, E1103, E1104, E1105, E1106, E1107, E1108, E1109, E1110, E1111, E1112, E1113, E1114, E1115, E1116, E1117, E1118, E1119, E1120, E1121, E1122, E1123, E1124, E1125, E1126, E1127, E1128, E1129, E1130, E1131, E1132, E1133, E1134, E1135, E1136, E1137, E1138, E1139, E1140, E1141, E1142, E1143, E1144, E1145, E1146, E1147, E1148, E1149, E1150, E1151, E1152, E1153, E1154, E1155, E1156, E1157, E1158, E1159, E1160, E1161, E1162, E1163, E1164, E1165, E1166, E1167, E1168, E1169, E1170, E1171, E1172, E1173, E1174, E1175, E1176, E1177, E1178, E1179, E1180, E1181, E1182, E1183, E1184, E1185, E1186, E1187, E1188, E1189, E1190, E1191, E1192, E1193, E1194, E1195, E1196, E1197, E1198, E1199
Obesity: E660, E6601, E6609, E661, E662, E668, E669, Z6830, Z6831, Z6832, Z6833, Z6834, Z6835, Z6836, Z6837, Z6838, Z6839, Z6841, Z6842, Z6843, Z6844, Z6845, 278, 2780, 27800, 27801, 27802, 27803
